# Maternal body mass index in pregnancy and mental disorders in adult offspring: a record linkage study in Aberdeen, Scotland

**DOI:** 10.1038/s41598-021-94511-y

**Published:** 2021-07-23

**Authors:** Marius Lahti-Pulkkinen, Katri Räikkönen, Sohinee Bhattacharya, Rebecca M. Reynolds

**Affiliations:** 1 Department of Psychology and Logopedics, Faculty of Medicine, University of Helsinki, Haartmaninkatu 3, 00014 Helsinki, Finland; 2 Institute of Applied Health Sciences, University of Aberdeen, Aberdeen, UK; 3 Centre for Cardiovascular Science and Tommy’s Centre for Fetal and Maternal Health, Queen’s Medical Research Institute, University of Edinburgh, Edinburgh, UK

**Keywords:** Psychology, Endocrinology, Risk factors, Medical research, Epidemiology

## Abstract

Maternal obesity in pregnancy predicts offspring psychopathology risk in childhood but it remains unclear whether maternal obesity or underweight associate with adult offspring mental disorders. We examined longitudinally whether maternal body mass index (BMI) in pregnancy predicted mental disorders in her offspring and whether the associations differed by offspring birth year among 68,571 mother–child dyads of Aberdeen Maternity and Neonatal Databank, Scotland. The offspring were born 1950–1999. Maternal BMI was measured at a mean 15.7 gestational weeks and classified into underweight, normal weight, overweight, moderate obesity and severe obesity. Mental disorders were identified from nationwide registers carrying diagnoses of all hospitalizations and deaths in Scotland in 1996–2017. We found that maternal BMI in pregnancy was associated with offspring mental disorders in a time-dependent manner: In offspring born 1950–1974, maternal underweight predicted an increased hazard of mental disorders [Hazard Ratio (HR) = 1.74; 95% Confidence Interval (CI) = 1.01–3.00)]. In offspring born 1975–1999, maternal severe obesity predicted increased hazards of any mental (HR 1.60; 95% CI 1.08–2.38) substance use (HR 1.91; 95% CI 1.03–3.57) and schizophrenia spectrum (HR 2.80; 95% CI 1.40–5.63) disorders. Our findings of time-specific associations between maternal prenatal BMI and adult offspring mental disorders may carry important public health implications by underlining possible lifelong effects of maternal BMI on offspring psychopathology.

## Introduction

Over recent decades, there has been a major change in the nutritional status of pregnant women. The incidence of obesity [body mass index(BMI) ≥ 30 kg/m^2^] has increased dramatically among women of childbearing age^1,2^, with prevalence rates of 7–25% in Europe and over 30% in the United States^1^. Epidemiological studies have consistently demonstrated a link between maternal undernutrition in pregnancy and adverse health consequences for the offspring^3^. However, increasing evidence shows that maternal obesity in pregnancy also predicts premature mortality, increased obesity, diabetes and cardiovascular disease risks and increased health care utilization in the offspring^4–9^. Although BMI is influenced also by several other factors than nutrition^10–12^, these findings can be seen as suggesting two developmental pathways of both maternal under- and over-nutrition being linked to offspring health.

Previous studies have shown that maternal overweight and obesity in pregnancy may also be associated with an increased risk of mental disorders and their symptoms in children^4,13–25^. A meta-analysis showed that maternal overweight (BMI 25–29.99 kg/m^2^) and obesity predicted increased risks of attention deficit hyperactivity disorder, autism spectrum disorder, and developmental delay in children^14^. To our knowledge, the largest study on maternal BMI and child mental disorders was a Finnish study among over 600 000 participants, which showed that children of women with obesity and particularly severe obesity (BMI ≥ 35 kg/m^2^) in early pregnancy had significantly increased risks of any mental disorder and several specific mental disorders^13,21^. However, almost all the studies included in the meta-analysis^14^ and the Finnish study^13,21^ followed-up the offspring only into childhood or adolescence. The exception was a Swedish study among over 300 000 participants, which followed-up offspring to ages 4–27 years, and found that offspring of women with overweight/obesity had increased risk of autism spectrum disorder, but not among a smaller subsample of differentially exposed siblings^18^.

Much less is known about the consequences of maternal underweight, overweight, obesity and severe obesity in pregnancy on offspring psychopathology risk in adulthood. The Dutch Hunger Winter and Chinese Famine studies showed that maternal undernutrition during pregnancy due to famine exposure predicted increased risks of schizophrenia, substance use, personality, and mood disorders in adult offspring^3,26–29^, but did not directly test effects of maternal BMI. The available data on maternal BMI in pregnancy and offspring adulthood psychopathology risk includes to our knowledge four studies, all on offspring schizophrenia, with between 336 and 10,500 participants and conflicting findings^30–33^. One study suggested a negative association of maternal BMI in pregnancy and offspring risk of schizophrenia^30^, two positive^31,33^, and one study showed no association^32^. However, none of these studies used the World Health Organization classification of BMI into categories^34^. Further, since the participants were born within a narrow range of birth years^30–33^, these studies could not test whether the effects of maternal BMI in pregnancy on offspring mental disorders are modified by offspring birth year, even though the rates of underweight, overweight, obesity and severe obesity have changed markedly across time^1^.

We hypothesized that both maternal underweight and obesity in pregnancy would be associated with an increased risk of offspring mental disorders in adulthood, with the greatest risk in the offspring of severely obese women^13^. To test this hypothesis, we examined the associations of maternal BMI in pregnancy and offspring mental disorders severe enough to require hospitalization or contribute to death in adulthood among 68,571 mother–offspring dyads identified from the Aberdeen Maternity and Neonatal Databank (AMND) and born 1950–1999. With such a range of birth years, we could examine the consequences of exposure to both maternal underweight and obesity in pregnancy, and test if the effects of maternal BMI on offspring mental disorders were time-dependent and varied by offspring birth year. We also tested if any associations were independent of several factors previously associated with maternal BMI and/or offspring mental disorders, including maternal mental disorders, maternal hypertensive pregnancy disorders and many sociodemographic and perinatal factors^13,18,23,24,35–37^.

## Results

### Sample characteristics

Table 1 shows the sociodemographic, maternal, and perinatal characteristics of our study sample. Table 2 shows the prevalence of any mental disorder and the specific mental disorder diagnostic categories in the offspring, along with the International Classification of Diseases, Tenth Revision codes used to identify these diagnoses and mean age at first diagnosis for each diagnosis. Mean age at first diagnosis of any mental disorder was 29.2 years, and 1103 (1.6%) offspring had been hospitalized or had died with a diagnosis of mental disorder. Mood disorders were the most common specific mental disorder category, followed by substance use and schizophrenia spectrum disorders.

**Table 1:** Characteristics of the whole study sample and of participants born 1950–1974 and 1975–1999.

	All participants			Participants born 1950–1974			Participants born 1975–1999		
	Data usable	Mean(SD)	N(%)	Data usable	Mean(SD)	N(%)	Data usable	Mean(SD)	N(%)
**Maternal characteristic**									
Body Mass Index	68,571	24.3(4.1)		9937	23.3(3.5)		58,634	24.5(4.2)	
Underweight			1546(2.3%)			321(3.2%)			1225(2.1%)
Normal weight			43,401(63.3%)			7253(73.0%)			36,148(61.7%)
Overweight			17,407(25.4%)			1879(18.9%)			15,528(26.5%)
Moderately obese			4577(6.7%)			397(4.0%)			4180(7.1%)
Severely obese			1640(2.4%)			87(0.9%)			1553(2.6%)
Weight(kg)	68,571	63.3(11.7)		9937	58.5(9.4)		58,634	64.1(11.8)	
Height(cm)	68,571	161.2(6.4)		9937	158.5(5.8)		58,634	161.7(6.3)	
Gestational week weight was measured	65,784	15.7 (7.1)		9935	(17.1(6.1)		55,849	15.5(7.2)	
< 20			53,814(81.8%)			7471(75.2%)			46,343(83.0%)
20–29			7894(12.0%)			2090(21.0%)			5804(10.4%)
30 or over			4076(6.2%)			374(3.8%)			3702(6.6%)
Hypertension in pregnancy	68,571			9937			58,634		
Normotension			50,255(73.3%)			7740(77.9%)			42,515(72.5%)
Pre-existing hypertension			644(0.9%)			0(NA)			644(1.1%)
Gestational hypertension			14,623(21.3%)			1825(18.4%)			12,798(21.8%)
Preeclampsia or eclampsia			3049(4.4%)			372(3.7%)			2677(4.6%)
Age at delivery	68,557	26.2(5.2)							
Smoking	50,565			2735			47,830		
Never smoked			29,102(57.6%)			1472(53.8%)			27,630(57.8%)
Quit smoking			2640(5.2%)			0(0.0%)			2640(5.5%)
Smoked throughout pregnancy			18,823(37.2%)			1263(46.2%)			17,560(36.7%)
Parity	68,571			9937			58,634		
Primiparous			46,657(68.0%)			5852(58.9%)			40,805(69.6%)
Other			21,914(32.0%)			4085(41.1%)			17,829(30.4%)
Register diagnosis of mental disorders	67,552			9149			58,403		
Yes			1892(2.8%)			525(5.7%)			1367(2.3%)
No			65,660(97.2%)			8624(94.3%)			57,036(97.7%)
Self-reported mental disorders by delivery	57,872			0			57,872		
Yes			2753(4.8%)			0			2753(4.8%)
No			55,119(95.2%)			0			55,119(95.2%)
**Family characteristics**									
Social deprivation: spouse’s occupation	49,311			8941			40,370		
Non manual			18,578(37.7%)			2013(22.5%)			16,565(41.0%)
Manual			30,733(62.3%)			6928(77.5%)			23,805(59.0%)
**Child characteristics**									
Sex	68,571			9937			58,634		
Girl			37,579(54.8%)			9074(91.3%)			28,505(48.6%)
Boy			30,992(45.2%)			863(8.7%)			30,129(51.4%)
Birth year	68,571	1984.1(11.8)		9937			58,634		
1950–1974			9937(15.5%)			9937(100%)			
1975–1999			58,634(85.5%)						58,634(100%)
Any mental disorder									
Yes	68,571		1103(1.6%)	9937		277(2.8%)	58,634		826(1.4%)
No			67,468(98.4%)			9660(97.2%)			57,808(98.6%)

All p-values for differences between the birth year groups in the maternal, perinatal and child characteristics < .001.

*SD* standard deviation; *NA* not available; *kg* kilogram; *cm* centimeter.

**Table 2:** The prevalence of and diagnostic codes for and the mean age at first diagnosis for the different offspring mental disorders in the whole cohort and among participants born 1950–1974 and 1975–1999.

Child mental disorder category	All participants			Participants born 1950–1974		Participants born 1975–1999	
	ICD-10 codes	N(%)	Age: Mean(SD)	N(%)	Age: Mean(SD)	N(%)	Age: Mean(SD)
Any mental disorder	F00-F99, X60-X84	1103(1.6%)	29.1(11.1)	277(2.8%)	43.7(9.9)	826(1.4%)	24.2(6.0)
Organic mental disorders	F0	17(0.0%)	42.0(16.4)	10(0.1%)	54.5(6.2)	7(0.0%)	24.1(5.1)
Substance use disorders	F1	371(0.5%)	29.9(10.3)	79(0.8%)	44.2(10.2)	292(0.5%)	26.0(6.1)
Schizophrenia, schizotypal and delusional disorders	F2	263(0.4%)	28.6(10.3)	62(0.6%)	42.5(10.2)	201(0.3%)	24.4(5.5)
Mood (affective) disorders	F3	469(0.7%)	30.5(11.4)	145(1.5%)	43.7(9.5)	324(0.6%)	24.7(5.9)
Anxiety (Neurotic, stress, somatoform) disorders	F4	182(0.3%)	29.8(10.9)	56(0.6%)	42.8(7.8)	126(0.2%)	24.0(6.1)
Other Behavioural syndromes	F5	50(0.1%)	23.7(8.3)	5(0.1%)	37.3(11.9)	45(0.1%)	22.2(6.3)
Disorders of adult personality and behavior	F6	110(0.2%)	28.1(9.8)	19(0.2%)	43.9(9.8)	91(0.2%)	24.8(5.7)
Mental retardation (intellectual disability)	F7	37(0.1%)	24.0(9.8)	7(0.1%)	39.3(13.0)	30(0.1%)	20.4(3.8)
Disorders of psychological development	F8	29(0.0%)	22.3(6.8)	NA	NA	28(0.0%)	21.8(6.5)
Emotional and behavioural disorders of childhood origin	F90-F98	11(0.0%)	17.3(5.3)	NA	NA	11(0.0%)	17.3(5.3)
Suicides	X60-X84	57(0.1%)	28.6(6.4)	7(0.1%)	44.2(7.6)	50(0.1%)	26.4(5.8)

N refers to the number and % to the percentage of cases with each diagnosis. ICD-10 codes refer to the International Classification of Diseases, Tenth Revision (ICD-10) diagnostic codes for each mental disorder diagnosis. Age refers to the age at first mental disorder diagnosis in years.

*SD* standard deviation.

Table 3 shows the associations of the covariates with offspring mental disorders. Maternal smoking during pregnancy, maternal physician-diagnosed mental disorders, younger maternal age, gestation when weight was measured, lower social class of the family, and earlier birth year of the child all showed significant associations with increased hazards of mental disorders in the offspring (Table 3, all p-values ≤ 0.02).

**Table 3:** The associations of the covariates with offspring hazard of mental disorders among all participants and participants born 1950–1974 and 1975–1999.

Covariate	All participants		Participants born 1950–1974		Participants born 1975–1999	
	Hazard ratio	p	Hazard ratio	p	Hazard ratio	p
	(95% confidence interval)		(95% confidence interval)		(95% confidence interval)	
**Maternal hypertension in pregnancy**						
Pre-existing hypertension vs. normal blood pressure	0.39 (0.15–1.05)	0.06	NA	NA	0.46 (0.17–1.23)	0.12
Gestational hypertension vs. normal blood pressure	1.08 (0.94–1.24)	0.29	0.85 (0.62–1.18)	0.34	1.19 (1.01–1.40)	0.03
Preeclampsia vs. normal blood pressure	1.27 (0.98–1.65)	0.07	0.93 (0.49–1.76)	0.83	1.43 (1.07–1.90)	0.02
**Maternal smoking during pregnancy**						
Quit vs. never smoked	0.99 (0.64–1.53)	0.96	NA	NA	1.07 (0.69–1.66)	0.75
Smoked Throughout vs. never smoked	2.73 (2.34–3.19)	< .001	1.94 (1.24–3.03)	0.004	2.80 (2.37–3.31)	< .001
**Gestation when maternal weight was measured**						
21–30 vs. 20 or less	1.49 (1.26–1.75)	< 0.001	1.38 (1.06–1.81)	0.02	1.32 (1.07–1.63)	0.01
31 or more vs. 20 or less	0.88 (0.67–1.17)	0.36	1.04 (0.55–1.96)	0.91	0.89 (0.66–1.21)	0.45
Maternal age at delivery	0.95 (0.94–0.97)	< 0.001	0.98 (0.95–1.00)	0.05	0.96 (0.94–0.97)	< 0.001
Parity: multiparous vs. primiparous	1.28 (1.13–1.44)	< 0.001	1.19 (0.94–1.51)	0.15	1.22 (1.06–1.41)	0.01
maternal hospital-treated mental disorders	3.47 (2.82–4.29)	< .001	1.28 (0.79–2.07)	0.31	4.47 (3.54–5.65)	< 0.001
Maternal self-reported mental disorders by delivery	1.39 (1.05–1.85)	0.02	NA	NA	1.39 (1.05–1.85)	0.02
Social class: manual vs. non-manual	1.49 (1.27–1.75)	< 0.001	1.54 (1.09–2.17)	0.01	1.28 (1.06–1.53)	0.01
Child sex: boy vs. girl	1.05 (0.93–1.18)	0.46	1.24 (0.84–1.82)	0.28	1.32 (1.15–1.52)	< 0.001
Child birth year	0.97 (0.96–0.97)	< 0.001	1.00 (0.99–1.02)	0.68	0.92 (0.91–0.93)	< 0.001
Child born 1975–1996 vs. 1950–1974	0.50 (0.44–0.57)	< 0.001	NA	NA	NA	NA

Hazard ratios and their 95% Confidence intervals refer to the hazard ratios and 95% confidence intervals of any mental disorder in the offspring.

*NA* not applicable.

### Time-specific associations

The associations between maternal BMI and offspring mental disorders were time-specific, which was shown by maternal BMI emerging as a time-dependent predictor of offspring mental disorders (p = 0.02). Hence, to meet the proportional hazards assumption, we ran all further analyses dividing our sample into two halves by birth year: participant offspring born in 1950–1974 and in 1975–1999. This yielded two subsamples of 9937 and 58,634 participants. Within these subsamples, the associations of maternal BMI with child mental disorders were not time-specific (p-values ≥ 0.13).

Tables 1, 2 and 3 also show the perinatal characteristics, prevalence of mental disorders, and associations of the covariates with mental disorders in these subsamples and the differences in these factors between the subsamples. The prevalence of maternal early pregnancy underweight decreased across time (2.1% in the younger vs. 3.2% in the older cohort, respectively), while there were more women with overweight (26.5% vs. 18.9%) and with moderate (7.1% vs. 4.0%) and severe (2.6% vs. 0.9%) obesity in early pregnancy in the younger cohort (Table 1). Mean age at first diagnosis of mental disorder was 43.7 years in the older cohort and 24.2 years in the younger subsample. Mood disorders, substance use disorders and schizophrenia spectrum disorders were the most common subcategories of mental disorders in both subsamples, each with average ages of onset in adulthood in both subsamples (Table 2). Children born 1950–1974 had higher hazards of mental disorders than children born 1975–1999 did (Table 3). Maternal age at childbirth, smoking during pregnancy, and hospital-treated mental disorders, gestation when maternal weight was measured, and lower social class of the family were each significantly associated with offspring mental disorders in both subsamples. Maternal preeclampsia, self-reported mental disorders and multiparity were associated with increased hazards of mental disorders among offspring born 1975–1999. Also among offspring born 1975–1999, boys and older participants had higher cumulative incidences of mental disorders (Table 3).

### Maternal BMI in pregnancy and offspring mental disorders among participants born 1950–1974

**Figure 1:**
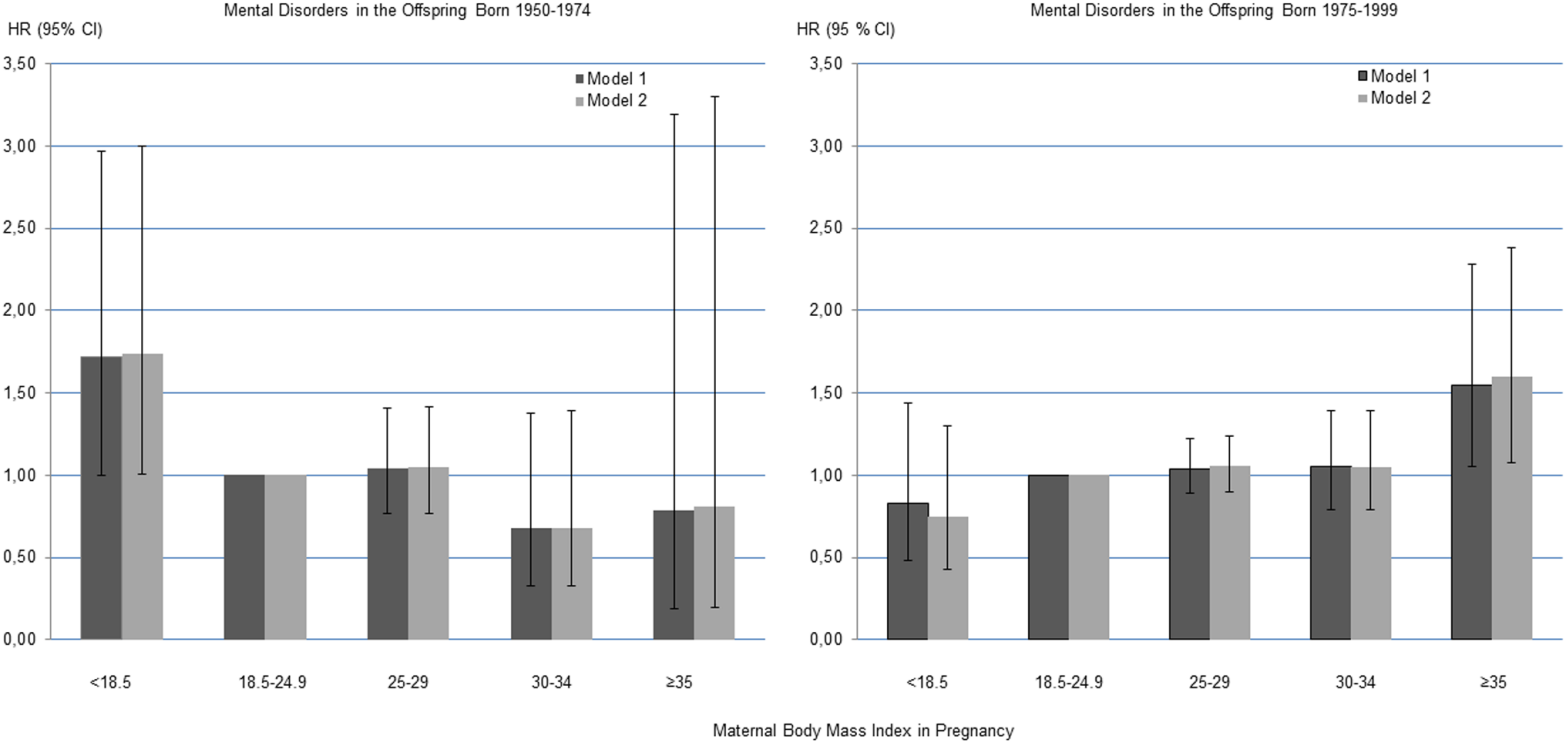
Maternal Body Mass Index in Pregnancy and Mental Disorders in the Offspring Born 1950–1974 and 1975–1999. Hazard Ratios (HR) and 95% Confidence Intervals (CI) from Cox Proportional Hazards Models adjusted for child sex and birth year and gestation at maternal weight measurement (Model 1) and additionally for maternal smoking and hypertensive disorders in pregnancy, maternal mental disorders, age, parity and social class of the family.

As shown in Table 4 and Fig. 1, in the older cohort, maternal underweight in pregnancy predicted a significantly (p = 0.05) increased, 1.7-fold hazard of any mental disorder in adult offspring. This association was independent of the birth year and sex of the child, gestational week at maternal weight measurement, maternal physician-diagnosed and self-reported mental disorders, hypertensive pregnancy disorders, smoking during pregnancy, age, parity, and social class of the family. Offspring of women with overweight, moderate obesity or severe obesity in pregnancy did not differ significantly in their hazards of mental disorders from offspring of women with normal weight in pregnancy in this subsample. None of the associations with offspring mental disorder subgroups was significant in this older subsample born 1950–1974.

**Table 4:** Maternal body mass index in pregnancy and hazard of mental disorders in offspring born 1950–1974.

Offspring mental disorder	Model 1		Model 2	
	Hazard ratio	p	Hazard ratio	p
	(95% confidence interval)		(95% confidence interval)	
**Any mental disorder**				
Underweight	1.72 (1.00–2.97)	0.05	1.74 (1.01–3.00)	0.05
Normal weight	Reference category			
Overweight	1.04 (0.77–1.41)	0.79	1.05 (0.77–1.42)	0.77
Moderate obesity	0.68 (0.33–1.38)	0.28	0.68 (0.33–1.39)	0.29
Severe obesity	0.79 (0.19–3.19)	0.74	0.81 (0.20–3.30)	0.77
**Substance use disorder**				
Underweight	1.25 (0.39–4.02)	0.70	1.19 (0.37–3.83)	0.77
Normal weight	Reference category			
Overweight	1.08 (0.62–1.90)	0.78	1.14 (0.64–2.01)	0.66
Moderate obesity	0.82 (0.25–2.68)	0.75	0.92 (0.28–3.03)	0.89
Severe obesity	1.32 (0.18–9.70)	0.78	1.74 (0.23–13.02)	0.59
**Schizophrenia spectrum disorders**				
Underweight	1.65 (0.51–5.35)	0.40	1.71 (0.53–5.55)	0.37
Normal weight	Reference category			
Overweight	0.82 (0.42–1.63)	0.58	0.77 (0.38–1.54)	0.46
Moderate obesity	0.61 (0.15–2.55)	0.50	0.54 (0.13–2.31)	0.41
Severe obesity	0 (NA)	0.98	0 (NA)	0.98
**Mood disorders**				
Underweight	1.72 (0.80–3.72)	0.17	1.71 (0.79–3.71)	0.17
Normal weight	Reference category			
Overweight	1.11 (0.74–1.66)	0.63	1.15 (0.76–1.74)	0.50
Moderate obesity	0.54 (0.17–1.72)	0.30	0.59 (0.18–1.89)	0.37
Severe obesity	0 (NA)	0.94	0 (NA)	0.94
**Anxiety disorders**				
Underweight	1.83 (0.56–5.96)	0.32	1.87 (0.57–6.10)	0.30
Normal weight	Reference category			
Overweight	1.74 (0.97–3.15)	0.06	1.74 (0.95–3.17)	0.07
Moderate obesity	0 (NA)	0.97	0 (NA)	0.96
Severe obesity	0 (NA)	0.98	0 (NA)	0.98

Model 1 is adjusted for the sex and birth year of the child and gestation when weight was measured. Model 2 is adjusted also for maternal smoking and hypertensive disorders in pregnancy, maternal hospital-treated and self-reported mental disorders, age at delivery, parity and the social class of the family.

*NA* not applicable.

### Maternal BMI in pregnancy and offspring mental disorders among participants born 1975–1999

Among offspring born 1975–1999, maternal severe obesity in early pregnancy was associated with a 1.6-fold significantly (p = 0.02) increased hazard of any mental disorder, and specifically 2.8-fold hazard of schizophrenia spectrum disorders and 1.9-fold hazard of substance use disorders (Table 5, Fig. 1), in the offspring. These associations were independent of all the covariates. Maternal moderate obesity or underweight showed no significant associations with offspring mental disorders. Maternal overweight was not associated with the hazard of any mental disorder in the offspring but maternal overweight in pregnancy did predict increased hazards of schizophrenia spectrum disorders and intellectual disability in the offspring.

**Table 5:** Maternal body mass index in pregnancy and hazard of mental disorders in offspring born 1975–1999.

Offspring mental disorder	Model 1		Model 2	
	Hazard ratio	p	Hazard ratio	p
	(95% confidence interval)		(95% confidence interval)	
**Any mental disorder**				
Underweight	0.83 (0.48–1.44)	0.51	0.75 (0.43–1.30)	0.30
Normal weight	Reference category			
Overweight	1.04 (0.89–1.22)	0.59	1.06 (0.90–1.24)	0.50
Moderate obesity	1.05 (0.79–1.39)	0.75	1.05 (0.79–1.39)	0.75
Severe obesity	1.55 (1.05–2.28)	0.03	1.60 (1.08–2.38)	0.02
**Substance use disorder**				
Underweight	0.73 (0.27–1.98)	0.54	0.61 (0.23–1.64)	0.33
Normal weight	Reference category			
Overweight	0.89 (0.68–1.18)	0.43	0.92 (0.70–1.21)	0.55
Moderate obesity	0.94 (0.58–1.54)	0.81	0.94 (0.57–1.54)	0.81
Severe obesity	1.80 (0.97–3.31)	0.06	1.91 (1.03–3.57)	0.04
**Schizophrenia spectrum disorders**				
Underweight	0.31 (0.04–2.22)	0.24	0.29 (0.04–2.06)	0.21
Normal weight	Reference category			
Overweight	1.42 (1.04–1.93)	0.03	1.47 (1.07–2.00)	0.02
Moderate Obesity	1.26 (0.72–2.20)	0.42	1.34 (0.76–2.37)	0.31
Severe Obesity	2.46 (1.24–4.87)	0.01	2.80 (1.40–5.63)	0.004
**Mood disorder**				
Underweight	1.15 (0.54–2.44)	0.72	1.06 (0.50–2.26)	0.87
Normal weight	Reference category			
Overweight	1.08 (0.84–1.39)	0.55	1.07 (0.83–1.38)	0.60
Moderate obesity	1.10 (0.70–1.71)	0.68	1.06 (0.67–1.66)	0.81
Severe obesity	1.52 (0.80–2.88)	0.20	1.46 (0.76–2.80)	0.25
**Anxiety disorder**				
Underweight	0.77 (0.19–3.14)	0.72	0.66 (0.16–2.70)	0.57
Normal weight	Reference category			
Overweight	1.00 (0.67–1.50)	0.99	1.03 (0.69–1.55)	0.88
Moderate obesity	0.58 (0.23–1.44)	0.24	0.57 (0.23–1.43)	0.23
Severe obesity	0.69 (0.17–2.84)	0.61	0.72 (0.17–2.99)	0.65
**Other behavioural disorder**				
Underweight	0 (NA)	0.97	0 (NA)	0.98
Normal weight	Reference category			
Overweight	1.24 (0.65–2.36)	0.51	1.24 (0.65–2.39)	0.51
Moderate obesity	0.35 (0.05–2.55)	0.30	0.36 (0.05–2.68)	0.32
Severe obesity	1.03 (0.14–7.65)	0.97	1.10 (0.15–8.30)	0.93
**Personality disorder**				
Underweight	0 (NA)	0.95	0 (NA)	0.95
Normal weight	Reference category			
Overweight	0.96 (0.58–1.57)	0.86	1.01 (0.61–1.66)	0.98
Moderate obesity	1.77 (0.92–3.43)	0.09	1.76 (0.90–3.44)	0.10
Severe obesity	0.97 (0.23–4.01)	0.97	1.03 (0.24–4.31)	0.97
**Intellectual disability**				
Underweight	2.13 (0.28–16.19)	0.47	1.88 (0.25–14.44)	0.54
Normal weight	Reference category			
Overweight	2.49 (1.16–5.31)	0.02	2.43 (1.13–5.23)	0.02
Moderate obesity	1.66 (0.38–7.33)	0.50	1.52 (0.34–6.81)	0.59
Severe obesity	0 (NA)	0.98	0 (NA)	0.98
**Suicide**				
Underweight	0 (NA)	0.96	0 (NA)	0.96
Normal weight	Reference category			
Overweight	1.06 (0.57–1.96)	0.85	1.15 (0.62–2.13)	0.67
Moderate obesity	0.27 (0.04–1.97)	0.19	0.32 (0.04–2.35)	0.26
Severe obesity	0.79 (0.11–5.86)	0.82	1.01 (0.14–7.59)	0.99

Model 1 is adjusted for sex and birth year of the child and gestation when weight was measured. Model 2 is adjusted also for maternal smoking and hypertensive disorders in pregnancy, maternal hospital-treated and self-reported mental disorders, age at delivery, parity and the social class of the family.

*NA* not applicable.

## Discussion

In our longitudinal cohort study, spanning over 60,000 births across five decades, maternal underweight and severe obesity in pregnancy emerged as time-dependent predictors of the hazard of mental disorders in adult offspring. While maternal underweight predicted increased hazard of any mental disorder in older offspring born in earlier years, among younger offspring, maternal severe obesity predicted increased offspring hazards of any mental disorder, substance use disorders, and schizophrenia spectrum disorders. All associations were independent of multiple covariates, including family’s social class, maternal mental disorders, hypertensive pregnancy disorders, and smoking in pregnancy. Our novel findings linking maternal severe obesity to offspring mental disorders also in adulthood are of particular concern given the rising prevalence of severe obesity among pregnant women^1^.

Few studies have examined effects of maternal underweight in pregnancy on offspring mental disorders. We found that in offspring born 1950–1974, offspring of women with underweight in early pregnancy had an increased risk of any mental disorder in adulthood. Our findings correspond with a Finnish study among offspring born 1924–1933 where lower maternal BMI predicted the risk of schizophrenia in adult offspring^30^, but not with a study in the United States among individuals born 1959–1967 which showed no effects of low maternal BMI on offspring schizophrenia risk^31^. These previous studies assessed maternal BMI at different gestational ages and with different methods^30,31^, and did not use the World Health Organization classification of underweight^34^. Also differences in the assessed mental health phenotypes and ages covered in diagnostic follow-ups may have contributed to the partially discrepant findings.

Notably, among participants born in 1975–1999, maternal severe prenatal obesity predicted increased hazards of any mental disorder and specifically substance use and schizophrenia spectrum disorders. These disorders most often had their onsets in young adulthood. Previous findings in children have also suggested that with increasing severity of maternal prenatal obesity, the consequences for offspring psychopathology risk may increase^13,19^. However, previous studies assessing effects of different levels of maternal obesity have had follow-up extending to adolescent age at most. The few available studies with follow-up extending to adulthood have not examined obesity severity effects, or focused on other mental disorders than schizophrenia or autism. In contrast to previous findings^13^, we found no effects of maternal moderate obesity on offspring mental disorders.

Importantly, the age specific findings in our study emerged particularly in those age groups where the exposure in question was more common: As shown in Table 1, maternal underweight was more common in the older and maternal severe obesity more common in the younger cohort. The incidence of underweight decreased and severe obesity increased across time in our cohort (Table 1). However, also the ages covered in the 20-year diagnostic follow-up differed depending on the birth year of the participant: participants born in the earlier years were older when the follow-up started, between 21 and 45 years of age in the older subsample and between 0 and 20 years old in the younger subsample. Hence, our time-specific findings may indicate either differential consequences of maternal BMI in pregnancy in different birth cohorts or at different ages.

Nevertheless, the more common forms of maternal abnormal BMI, possibly indicating malnutrition, observed among pregnant women across the world, were the key predictors of offspring mental ill health: underweight maybe indicating undernutrition in the older, earlier-born cohort, and severe obesity possibly signifying over-nutrition in the younger cohort. These observations chime with the Developmental Origins of Health and Disease—framework of chronic illnesses, according to which prenatal adversity leads to permanent changes in the development of organs, cells, and body’s biological feedback mechanisms, thereby contributing to an increased risk of chronic illnesses, including mental disorders subsequently^38^. Supporting the DOHaD framework and the possible role of under-or overnutrition underlying our findings, previous studies have shown that maternal undernutrition during pregnancy due to famine exposure may predict the risks of several different mental disorders in the offspring^3,26,27,29^, that specific maternal dietary patterns may predict increased risks of psychiatric symptoms and suboptimal neurodevelopment in children^25^, and that maternal obesity, dietary patterns and undernutrition in pregnancy may each predict altered brain structure in the offspring^3,25,39^.

However, also other mechanisms than maternal nutrition may have contributed to our findings. Convincing evidence shows that maternal obesity is associated with increased depressive and symptoms during pregnancy^40^ and that maternal depressive and anxiety symptoms during pregnancy are associated with increased psychopathology risk in the offspring^41,42^. Increased psychological distress during pregnancy in obese women, contributing to offspring psychopathology risk via prenatal programming or genetic or shared environmental pathways^43^ may thus explain the associations between maternal BMI and offspring mental disorders. Furthermore, lower socioeconomic position is consistently associated with an increased risk of obesity^12^, and previous evidence shows that there is a clear socioeconomic ingredient also in the AMND for the prevalence of maternal early pregnancy obesity both in the older^44^ and younger^45^ birth cohorts for the current study. As lower childhood socioeconomic position predicts an increased risk of mental disorders^24^, also in our study sample (Table 3), adverse childhood socioeconomic circumstances offer an additional pathway possibly contributing to our findings. However, neither childhood social class, as indicated by deprivation level of the family, nor maternal mental disorders explained the associations we found between maternal BMI with offspring psychopathology. On the other hand, maternal exposure to childhood maltreatment may also have contributed: Exposure to childhood maltreatment is associated with obesity risk^11^, also in pregnant women^46^, and childhood maltreatment predicts increased psychopathology risk both within the same^47^ and the next^48,49^ generation. A Mendelian randomization study suggests the effects on mental health within the same generation may be causal^50^. It is here of note that a large proportion of the mothers in the older subsample of the current study were children during World War II. Since Aberdeen was during the war affected by numerous air raids and had a large number of civilian victims, the childhood circumstances may have been adverse and traumatic for many women of our cohort. While we did not have data on maternal childhood maltreatment or other childhood traumas in our cohort, further studies should more specifically examine and compare the role of different etiological factors to the associations between maternal BMI in pregnancy and offspring mental disorders.

Neurobiologically, inflammatory pathways and other mechanisms related to individual’s altered stress vulnerability because of maternal obesity or underweight may have contributed to our findings. Obesity in pregnancy is a highly proinflammatory state^4,17,25,51^, and prenatal inflammation has been associated with psychopathology risk in the offspring^17,52,53^. Maternal BMI in pregnancy has been associated with altered functioning of the hypothalamus-pituitary adrenal axis in the offspring^54^, and changes in the functioning of this axis are characteristic of patients with mental disorders^55^. Furthermore, shared genetic vulnerabilities contributing both to our predictor and outcome phenotypes^10,56^ and/or maternal underweight or obesity in pregnancy leading to epigenetic changes in gene expression and contributing to an increased risk of mental disorders in the offspring^4,57,58^ are among the possible molecular pathways underlying the associations of maternal BMI in pregnancy with offspring mental disorders.

Our study has several strengths, including the longitudinal study design covering a combination of the whole range of mental disorders as outcomes and a lengthy follow-up extending into and/or across adulthood and over a period of time with dramatic changes in maternal BMI during pregnancy. Such a design has to our knowledge not been used in previous studies on maternal BMI and offspring psychopathology. With births spanning across five decades, we were able to assess temporal changes in the effects of maternal BMI in pregnancy on offspring mental disorders. We also benefited from a representative sample of over 60,000 participants. Due to the rich dataset, we could show effects occurring independently of maternal mental disorders, social class of the family, and the highly comorbid maternal hypertensive pregnancy disorders. When examining the associations of maternal BMI in pregnancy and child mental disorders, many studies have adjusted for maternal self-reported psychopathology, but fewer have controlled for physician-diagnosed mental disorders identified from nationwide health registers. Corresponding to the scarce previous evidence^13,15,16,18,21^, we found that maternal BMI in pregnancy predicted offspring psychopathology risk independently of maternal mental disorders and added to the previous findings by showing that such effects persisted to adulthood.

The limitations of our study included having no data on paternal BMI or mental health-the only paternal factor available was social class. Such paternal data would be important to disentangle prenatal programming effects from familial confounding. Unfortunately, register data in most countries lack such data on paternal factors during the offspring’s prenatal period. Furthermore, in the older subsample of our cohort, female offspring were overrepresented. This is most likely due to the more thorough BMI data available for the women of the cohort due to other AMND studies assessing the intergenerational transmission of perinatal characteristics through the maternal line^2^. Furthermore, we lacked data on maternal diabetes which often co-occurs with maternal obesity^1,4,13^. Indeed, previous evidence suggests that maternal diabetes and severe obesity may have additive effects on child mental disorders^13^, and further studies should examine if such additive effects extend to adult age. As outcomes, we focused on mental disorders severe enough to require hospitalization or contribute to death. It is thus questionable whether our findings generalize to less severe forms of psychopathology. We had diagnostic data only from Scotland, and were unable to identify whether participants had moved abroad during the follow-up. These factors may have biased our analyses. Also, since the distribution of maternal BMI differed between the subsamples of our study, statistical power to detect associations was stronger in the subsamples where the exposure in question was more common, which could suggest that bias related to differential statistical power would have contributed to the discrepant findings in the subsamples. Nevertheless, the significant time-dependency of the effects of maternal BMI on offspring mental disorders, and the clearly different hazard ratios in the maternal BMI groups within these subsamples do point out towards time- and/or birth-cohort-specific associations of maternal BMI with offspring mental disorders in the AMND. Also, a potential bias may stem from the youngest participating offspring in our younger cohort having a shorter diagnostic follow-up than the other cohort members, since they were born during the follow-up period. However, we adjusted all our analysis for offspring birth year. While most often the mental disorders in our cohort were first diagnosed in adulthood, in the younger subsample a smaller proportion of the cohort had received their first diagnosis in childhood or adolescence. Nevertheless, the relatively rare childhood onset emotional and behavioral disorders were the only diagnostic category with average age of onset before 20 years, while all other diagnostic categories had their average age at first diagnosis in adult age. Therefore, our findings are best generalized to mental disorders in adulthood. Furthermore, although we focused on the first pregnancies of the mothers in the cohort to exclude statistical bias due to dependent observations, this led to an overrepresentation of primiparous mothers in our sample. Since maternal parity was here and has been previously associated with offspring psychopathology risk^36^, further studies should examine whether similar or different associations between maternal underweight or severe obesity and offspring mental disorders emerge in more multiparous mothers and their children.

In conclusion, in our large prospective study, we found birth cohort specific effects of maternal BMI in pregnancy on offspring mental disorders. Among older participants born in earlier years, adult offspring of women with underweight in pregnancy had increased hazards of mental disorders, while among participants born more recently and in their young adulthood during our diagnostic follow-up, maternal severe obesity in pregnancy predicted increased hazards of any mental, schizophrenia spectrum, and substance use disorders. Our findings are important due to both the large numbers of women with underweight in pregnancy, and the increasing number of women with obesity in pregnancy. The findings highlight the potential permanent effects of maternal BMI on offspring psychopathology risk across the lifespan.

## Methods

Our study cohort, the AMND, comprises all births at the Aberdeen Maternity Hospital since 1950^59^. The database includes pregnancy, delivery and baby records on obstetric and perinatal health. Since around 99% of the births in the city occur at the Aberdeen Maternity Hospital^59^, the AMND is representative of the whole Aberdeen population.

For the current study, we had valid data on maternal BMI in pregnancy and child mental disorders for 68,571 mothers and their children born 1950–1999. We restricted our analyses to the participants who were alive during the Scottish Morbidity Records diagnostic follow-up between January 1, 1996 and June 8, 2017 and could hence have received a mental disorder diagnosis. Since BMI shows high consistency across adulthood, we focused on the 68,571 first live-born offspring of the mothers in the dataset in order to have independent analytic observations and meet the assumptions of the statistical analyses conducted^31^.

The authors assert that all procedures contributing to this work comply with the ethical standards of the relevant national and institutional committees on human experimentation and with the Helsinki Declaration of 1975, as revised in 2008. Our study was completely a record linkage study, and hence informed consent was unnecessary and impossible to obtain. We did not have identification information for the cohort members and some of them are already deceased. We did not conduct active experiments for our cohort members. Rather, we have used maternity, neonatal and health register data from a wide cohort where national register authorities have conducted the necessary data linkages. All procedures have been in place to protect the privacy of the participants. In Scottish nationwide health register studies, the researchers ask DASH Safehaven Scotland to provide a dataset linking the data from different registers. This linked dataset uses a pseudonymized study id code to link the data, and it is impossible for the researchers to identify the cohort members and ask for consents. This is an important prerequisite for record linkage studies to protect the privacy of the cohort members, since the study data is sensitive. All procedures involving human subjects were approved by the NHS Scotland's Public Benefit and Privacy Panel for Health and Social Care (Reference 516–0594) and the AMND steering committee. Thus, informed consent is also waived by the ethics committees NHS Scotland's Public Benefit and Privacy Panel for Health and Social Care (Reference 516-0594) and the AMND steering committee.

### Maternal BMI

Maternal BMI was calculated from weight and height measured at first antenatal booking, on average at 15.1 gestational weeks, and extracted from the AMND register dataset. While over 80% of mothers had their weight and height measured in the first 20 gestational weeks, there was variation in the gestational stage when weight was measured. Hence, we adjusted all analyses for the time of weight measurement, classified into groups of < 20-weeks, = 20–30 weeks, and ≥ 31 weeks. We classified maternal BMI into five categories according to the World Health Organization classification: women with underweight < 18.5, normal weight = 18.5–24.9, overweight = 25.0–29.9, moderate (class 1) obesity = 30.0–34.9 and severe (class ≥ 2) obesity ≥ 35 in early pregnancy^34^.

### Mental disorders in mothers and children

For all AMND mothers and children, mental disorder diagnoses were extracted from the Scottish Morbidity Records and National Records of Scotland^59^. These registers carry International Classification of Diseases, Tenth Revision diagnoses for all hospitalizations and causes of death in Scotland between January 1, 1996 and June 8, 2017. The datasets have complete nationwide coverage for this period. The diagnostic follow-up started at birth for the youngest and at 45 years of age for the oldest participant offspring. At the end of the follow-up, the offspring were 17–67 years old. The data linkage between the registers was done with deterministic matching using Community Health Index where available and with probabilistic matching using name, surname, date of birth, sex, and postcode when the Community Health Index was unavailable^59^.

We identified maternal and child mental disorders with International Classification of Diseases, Tenth Revision diagnostic codes F00-F99 (mental and behavioral disorders) and X60-X84 (suicides). We assessed associations of maternal BMI in pregnancy with any mental disorder in the offspring and with the two-digit International Classification of Diseases diagnostic groups with at least 30 participants diagnosed with the mental disorder in question during the follow-up. This cutoff was used to meet the requirements for reliable Cox Regression analyses and to ensure the anonymity of the participants. We also excluded mental disorder categories with only one participant from descriptive tables to certify that no participants could be identified.

Maternal physician-diagnosed mental disorders were used as a binary covariate. This variable indicated whether the mother had been hospitalized or had died with a diagnosis of mental disorder.

### Maternal, sociodemographic and perinatal covariates

We identified the following covariates from the AMND: maternal hypertensive pregnancy disorders, smoking during pregnancy, self-reported mental disorders by childbirth, age at delivery and parity, mother’s partner’s deprivation category and child sex and birth year. These covariates were chosen based on their previously reported associations with maternal BMI in pregnancy in AMND^6,44,45^ and with psychopathology risk in the offspring^24,35–37,60^, and on them completing the epidemiologic research and causal diagram criteria for possible confounders being pre-exposure or other non-descendent factors related to the exposure (and outcome)^61^.

Women with self-reported mental disorders by childbirth were identified with questions on whether the mother had been diagnosed with depressive, anxiety, somatoform, or dissociative disorders, unspecified psychosis, or perinatal mental disorders, or been prescribed antipsychotics. We used a binary variable indicating the presence or absence of any of these psychiatric problems, recorded in the AMND.

The family’s socioeconomic position was defined based on the deprivation category of the partner of the mother. This index is based on living area and assesses the amount of social deprivation in the area on a scale from one to six. Values 1–3 refer to areas predominated by non-manual and values 4–6 by manual occupations.

Maternal age (years) at childbirth was used as a continuous covariate. Parity was classified into two categories (primiparous vs. other). Since we included the first-born offspring of the mothers in the AMND dataset with diagnostic data in 1996–2017 to the study, our sample had a high prevalence of primiparous women but also comprised 21,917 multiparous women.

We classified maternal smoking during pregnancy into three categories: never smoked, quit smoking during pregnancy or smoked throughout pregnancy. We classified maternal hypertensive pregnancy disorders into four categories: Normal blood pressure, pre-existing/essential hypertension, gestational hypertension, and preeclampsia/eclampsia.

Participants with missing values on categorical covariates were classified into their own respective categories. For maternal age, we imputed mean values for the 14 participants with missing data.

### Statistical analyses

We examined the associations of the covariates with offspring mental disorders with Cox Proportional Hazards models. The participants were followed up from January 1, 1996 to June 8, 2017 or their death and censored from the analyses on these dates or the date they were hospitalized for mental disorders.

We examined the associations of maternal BMI in pregnancy with mental disorders in the offspring also using Cox models. We contrasted offspring born to underweight, overweight, moderately and severely obese women with offspring of normal weight women. First analytic models were adjusted for the birth year and sex of the child and gestational week at maternal weight measurement. Second models adjusted also for maternal physician-diagnosed and self-reported mental disorders, hypertensive pregnancy disorders, smoking during pregnancy, age, parity, and social class of the family.

Before proceeding to the above analyses, we studied if the birth year of the child modified the effects of maternal BMI in pregnancy on child mental disorders. We examined this by testing if maternal BMI had time-dependent effects on offspring mental disorders, using maternal BMI as a time-dependent covariate in the Cox models.

## Data Availability

Due to the sensitive nature of the patient diagnostic data gathered for the current study, our data cannot be made open access. The datasets generated and analyzed during the current study are not publicly available due to prohibitions by national laws since the data include patient data. Data requests for AMND data require specific data and ethical approval requests by the AMND Steering Committee^59^.

## References

[1] Poston L (2016). Preconceptional and maternal obesity: Epidemiology and health consequences. Lancet Diabetes Endocrinol..

[2] Lahti-Pulkkinen M (2018). Intergenerational transmission of birth weight across 3 generations. Am. J. Epidemiol..

[3] Roseboom TJ (2019). Epidemiological evidence for the developmental origins of health and disease: Effects of prenatal undernutrition in humans. J. Endocrinol..

[4] Godfrey KM (2017). Influence of maternal obesity on the long-term health of offspring. Lancet Diabetes Endocrinol..

[5] Lahti-Pulkkinen M (2019). Consequences of being overweight or obese during pregnancy on diabetes in the offspring: A record linkage study in Aberdeen, Scotland. Diabetologia.

[6] Reynolds RM (2013). Maternal obesity during pregnancy and premature mortality from cardiovascular event in adult offspring: Follow-up of 1 323 275 person years. BMJ.

[7] Heslehurst N (2019). The association between maternal body mass index and child obesity: A systematic review and meta-analysis. PLoS Med..

[8] Kuhle S (2019). Maternal pre-pregnancy obesity and health care utilization and costs in the offspring. Int. J. Obes..

[9] Magnus MC (2018). Paternal and maternal obesity but not gestational weight gain is associated with type 1 diabetes. Int. J. Epidemiol..

[10] Amare AT, Schubert KO, Klingler-Hoffmann M, Cohen-Woods S, Baune BT (2017). The genetic overlap between mood disorders and cardiometabolic diseases: A systematic review of genome wide and candidate gene studies. Transl. Psychiatry.

[11] Hemmingsson E, Johansson K, Reynisdottir S (2014). Effects of childhood abuse on adult obesity: A systematic review and meta-analysis. Obes. Rev..

[12] El-Sayed AM, Scarborough P, Galea S (2012). Unevenly distributed: A systematic review of the health literature about socioeconomic inequalities in adult obesity in the United Kingdom. BMC Public Health.

[13] Kong L, Norstedt G, Schalling M, Gissler M, Lavebratt C (2018). The risk of offspring psychiatric disorders in the setting of maternal obesity and diabetes. Pediatrics.

[14] Sanchez CE (2017). Maternal pre-pregnancy obesity and child neurodevelopmental outcomes: A meta-analysis. Obes. Rev..

[15] Musser ED (2017). Maternal prepregnancy body mass index and offspring attention-deficit/hyperactivity disorder: A quasi-experimental sibling-comparison, population-based design. J. Child Psychol. Psychiatry Allied Discip..

[16] Grudzinski A (2019). Maternal pre-pregnancy weight status and health care use for mental health conditions in the offspring. Eur. Child Adolesc. Psychiatry.

[17] van der Burg JW (2016). The role of systemic inflammation linking maternal BMI to neurodevelopment in children. Pediatr. Res..

[18] Gardner RM (2015). Maternal body mass index during early pregnancy, gestational weight gain, and risk of autism spectrum disorders: Results from a Swedish total population and discordant sibling study. Int. J. Epidemiol..

[19] Jo H, Schieve LA, Sharma AJ, Hinkle SN, Li R (2015). Maternal prepregnancy body mass index and child psychosocial development at 6 years of age. Pediatrics.

[20] Mina TH (2017). Prenatal exposure to very severe maternal obesity is associated with adverse neuropsychiatric outcomes in children. Psychol. Med..

[21] Kong L, Nilsson IAK, Brismar K, Gissler M, Lavebratt C (2020). Associations of different types of maternal diabetes and body mass index with offspring psychiatric disorders. JAMA Netw. Open.

[22] Rodriguez A (2008). Maternal adiposity prior to pregnancy is associated with ADHD symptoms in offspring: Evidence from three prospective pregnancy cohorts. Int. J. Obes..

[23] Neuhaus ZF (2020). Maternal obesity and long-term neuropsychiatric morbidity of the offspring. Arch. Gynecol. Obstet..

[24] Lahti-Pulkkinen M (2020). Maternal hypertensive pregnancy disorders and mental disorders in children. Hypertension.

[25] Hasebe K, Kendig MD, Morris MJ (2021). Mechanisms underlying the cognitive and behavioural effects of maternal obesity. Nutrients.

[26] Brown AS, Susser ES (2008). Prenatal nutritional deficiency and risk of adult schizophrenia. Schizophr. Bull..

[27] Neugebauer R, Hoek HW, Susser E (1999). Prenatal exposure to wartime famine and development of antisocial personality disorder in early adulthood. J. Am. Med. Assoc..

[28] Wu L (2017). Prenatal exposure to the Great Chinese Famine and mid-age hypertension. PLoS ONE.

[29] Franzek EJ, Sprangers N, Janssens ACJW, Van Duijn CM, Van De Wetering BJM (2008). Prenatal exposure to the 1944–45 Dutch ‘hunger winter’ and addiction later in life. Addiction.

[30] Wahlbeck K, Forsen T, Osmond C, Barker DJP, Eriksson JG (2001). Association of schizophrenia with low maternal body mass index, small size at birth, and thinness during childhood. Arch. Gen. Psychiatry.

[31] Schaefer CA (2000). Maternal prepregnant body mass and risk of schizophrenia in adult offspring. Schizophr. Bull..

[32] Jones PB (1998). Schizophrenia as a long-term outcome of pregnancy, delivery and perinatal complications: A 28 year follow-up of the 1966 North Finland general population birth cohort. Am. J. Psychiatry.

[33] Kawai M (2004). Poor maternal care and high maternal body mass index in pregnancy as a risk factor for schizophrenia in offspring. Acta Psychiatr. Scand..

[34] World Health Organization (2000). Obesity: Preventing and managing the global epidemic. Report of a WHO consultation. WHO Tech. Rep. Ser..

[35] Ekblad M, Lehtonen L, Korkeila J, Gissler M (2017). Maternal smoking during pregnancy and the risk of psychiatric morbidity in singleton sibling pairs. Nicotine Tob. Res..

[36] Lahti M (2014). Maternal grand multiparity and the risk of severe mental disorders in adult offspring. PLoS ONE.

[37] Maher GM (2020). Association between preeclampsia and attention-deficit hyperactivity disorder: A population-based and sibling-matched cohort study. Acta Psychiatr. Scand..

[38] Barker DJP (2007). The origins of the developmental origins theory. J. Intern. Med..

[39] Verdejo-Román J (2019). Maternal prepregnancy body mass index and offspring white matter microstructure: Results from three birth cohorts. Int. J. Obes..

[40] Dachew BA, Ayano G, Betts K, Alati R (2021). The impact of pre-pregnancy BMI on maternal depressive and anxiety symptoms during pregnancy and the postpartum period: A systematic review and meta-analysis. J. Affect. Disord..

[41] Tuovinen S (2021). Maternal antenatal stress and mental and behavioral disorders in their children. J. Affect. Disord..

[42] Madigan S (2018). A meta-analysis of maternal prenatal depression and anxiety on child socio-emotional development. J. Am. Acad. Child Adolesc. Psychiatry.

[43] Jami, E. S., Hammerschlag, A. R., Bartels, M. & Middeldorp, C. M. Parental characteristics and offspring mental health and related outcomes: A systematic review of genetically informative literature. *Transl. Psychiatry* **11** (2021). 10.1038/s41398-021-01300-2 PMC8016911 33795643

[44] Lee KK (2015). Maternal obesity during pregnancy associates with premature mortality and major cardiovascular events in later life. Hypertension.

[45] Bhattacharya S, Campbell DM, Liston WA, Bhattacharya S (2007). Effect of Body Mass Index on pregnancy outcomes in nulliparous women delivering singleton babies. BMC Public Health.

[46] Leonard SA, Petito LC, Rehkopf DH, Ritchie LD, Abrams B (2017). Maternal history of child abuse and obesity risk in offspring: Mediation by weight in pregnancy. Child. Obes..

[47] Li M, D’Arcy C, Meng X (2016). Maltreatment in childhood substantially increases the risk of adult depression and anxiety in prospective cohort studies: Systematic review, meta-analysis, and proportional attributable fractions. Psychol. Med..

[48] Plant DT, Jones FW, Pariante CM, Pawlby S (2017). Association between maternal childhood trauma and offspring childhood psychopathology: Mediation analysis from the ALSPAC cohort. Br. J. Psychiatry.

[49] Su Y, D’Arcy C, Meng X (2020). Intergenerational effect of maternal childhood maltreatment on next generation’s vulnerability to psychopathology: A systematic review with meta-analysis. Trauma Violence Abus..

[50] Warrier V (2021). Gene–environment correlations and causal effects of childhood maltreatment on physical and mental health: A genetically informed approach. The Lancet Psychiatry.

[51] Lahti-Pulkkinen M (2020). Maternal depression and inflammation during pregnancy. Psychol. Med..

[52] Girchenko P (2020). Persistently high levels of maternal antenatal inflammation are associated with and mediate the effect of prenatal environmental adversities on neurodevelopmental delay in the offspring. Biol. Psychiatry.

[53] Zhang J, Luo W, Huang P, Peng L, Huang Q (2018). Maternal C-reactive protein and cytokine levels during pregnancy and the risk of selected neuropsychiatric disorders in offspring: A systematic review and meta-analysis. J. Psychiatr. Res..

[54] Kumpulainen SM (2019). Maternal early pregnancy body mass index and diurnal salivary cortisol in young adult offspring. Psychoneuroendocrinology.

[55] Zorn JV (2017). Cortisol stress reactivity across psychiatric disorders: A systematic review and meta-analysis. Psychoneuroendocrinology.

[56] van den Broek N (2018). Causal associations between body mass index and mental health: A Mendelian randomisation study. J. Epidemiol. Community Health.

[57] Barker ED, Walton E, Cecil CAM (2018). Annual research review: DNA methylation as a mediator in the association between risk exposure and child and adolescent psychopathology. J. Child Psychol. Psychiatry.

[58] Sharp GC (2017). Maternal BMI at the start of pregnancy and offspring epigenome-wide DNA methylation: Findings from the pregnancy and childhood epigenetics (PACE) consortium. Hum. Mol. Genet..

[59] Ayorinde AA, Wilde K, Lemon J, Campbell D, Bhattacharya S (2016). Data resource profile: The Aberdeen Maternity and Neonatal Databank (AMND). Int. J. Epidemiol..

[60] Wiles NJ, Peters TJ, Leon DA, Lewis G (2005). Birth weight and psychological distress at age 45–51 years: Results from the Aberdeen Children of the 1950s cohort study. Br. J. Psychiatry.

[61] Vander Weele TJ, Shpitser I (2013). On the definition of a confounder. Ann. Stat..

